# Using machine‐learning approach to distinguish patients with methamphetamine dependence from healthy subjects in a virtual reality environment

**DOI:** 10.1002/brb3.1814

**Published:** 2020-08-29

**Authors:** Xinfang Ding, Yuanhui Li, Dai Li, Ling Li, Xiuyun Liu

**Affiliations:** ^1^ School of Medical Humanities Capital Medical University Beijing China; ^2^ Adai Technology (Beijing) Ltd., Co Beijing China; ^3^ School of Computing University of Kent Kent UK; ^4^ Department of Anesthesiology and Critical Care Medicine School of Medicine Johns Hopkins University Baltimore MD USA; ^5^ School of Precision Instrument and Optoelectronics Engineering Tianjin University Tianjin China

**Keywords:** drug abuse, electroencephalography, machine learning, methamphetamine, virtual reality

## Abstract

**Background:**

The aim of this study was to evaluate whether machine learning (ML) can be used to distinguish patients with methamphetamine dependence from healthy controls by using their surface electroencephalography (EEG) and galvanic skin response (GSR) in a drug‐simulated virtual reality (VR) environment.

**Methods:**

A total of 333 participants with methamphetamine (METH) dependence and 332 healthy control subjects were recruited between January 2018 and January 2019. EEG (five electrodes) and GSR signals were collected under four VR environments: one neutral scenario and three METH‐simulated scenarios. Three ML classification techniques were evaluated: random forest (RF), support vector machine (SVM), and logistic regression (LR).

**Results:**

The MANOVA showed no interaction effects among the two subject groups and the 4 VR scenarios. Taking patient groups as the main effect, the METH user group had significantly lower GSR, lower EEG power in delta (*p* < .001), and alpha bands (*p* < .001) than healthy subjects. The EEG power of beta band (*p* < .001) and gamma band (*p* < .001) was significantly higher in METH group than the control group. Taking the VR scenarios (*Neutral* versus *METH‐VR*) as the main effects, the GSR, EEG power in delta, theta, and alpha bands in neutral scenario were significantly higher than in the METH‐VR scenario (*p* < .001). The LR algorithm showed the highest specificity and sensitivity in distinguishing methamphetamine‐dependent patients from healthy controls.

**Conclusion:**

The study shows the potential of using machine learning to distinguish methamphetamine‐dependent patients from healthy subjects by using EEG and GSR data. The LR algorithm shows the best performance comparing with SVM and RF algorithm.

## Graphical abstract

The study shows the potential of using machine learning to distinguish drug‐addicted patients from healthy subjects by using surface EEG and galvanic skin response data. The random forest algorithm shows the best performance comparing with support vector machine and logistic regression.

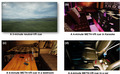

## 1 INTRODUCTION

Substance dependence brings serious problems to the society, including disease, crime, accidents, domestic violence, homelessness, etc. One in four deaths and almost 80% of domestic violence crimes were caused by alcohol abuse, smoking, and illegal drug use (Horgan & Strickler, 2001). Among all types of drugs, methamphetamine (METH) is considered as one of the biggest threats. According to the 2017 China Drug Use Report (Commission O of CNNC, 2017), an estimated 2.55 million Chinese people had used drugs illegally, 80 percent of which were male (Cai, Gao, & Wang, 2017). Substance dependence disorders are chronically relapsing disorders and a chronic health condition. Cognitive processing of drug‐related cues (e.g., glass pipe, medical tubing) and the subsequent dysregulation of behavior play a critical role in the relapse. Therefore, it is important to identify the neural correlate pattern of drug‐related cues in the patients with substance dependence. It has been proved that the brain of patients with substance dependence disorders presents altered structure and neurophysiological abnormalities (Cai et al., 2017; Coullaut‐Valera et al., 2014; Prichep et al., 1999; Turnip et al., 2017). Electroencephalography (EEG) is one of the available tools for examining the effects of drugs on brain function. Some investigations indicated that drug‐dependent individuals had more significant responses to drug‐related stimuli than control group by examining EEG responses evoked by cocaine‐relevant and cocaine‐irrelevant stimuli (Van De Laar, Licht, Franken, & Hendriks, 2004); some researchers found that the high craving group showed a larger positive slow wave compared to the low craving group following the presentation of cocaine‐related pictures (Franken, Hulstijn, Stam, Hendriks, & Van Den Brink, 2004). With the development of modern computational techniques, machine learning (ML) has been applied in various fields, which mainly serves two purposes: classifying and predicting and are divided into supervised and unsupervised algorithms (Sakr et al., 2017). Distinguishing normal and patients with methamphetamine dependence through EEG using ML has the advantage of wide availability, relatively low‐cost, easy implementation, and noninvasiveness. In the present study, we evaluated and compared the accuracy of distinguishing patients with methamphetamine dependence and healthy control subjects of three popular supervised ML algorithms based on their EEG and galvanic skin response (GSR) data. In particular, we conducted experiments under a virtual reality (VR) environment as suggested by Culbertson et al. (2010). Three ML techniques were compared: support vector machine (SVM), random forest (RF), and logistic regression (LR).

## 2 MATERIALS AND METHODS

### 2.1 Participants

Three hundred and thirty‐three participants with methamphetamine (METH) dependence were recruited between January 2018 and January 2019 admitted to Jidong drug rehabilitation center located in Shandong, China. This rehabilitation institution is for males only, which accommodates over 1,000 drug users and patients inside the institution stay completely abstinent from drugs. The center provides medical treatment for physical problems; however, no medical or psychological interventions that target drug abuse are provided. The METH users are arranged to do some daily activities (e.g., reading books, handcraft, etc.) when they were in the institution. Written consent forms were obtained from all the participants. The data analysis was approved by the local review board (IRB). Personal data and history of drug use were recorded by the experimenter. The inclusion criteria were as follows: (a) diagnosed with drug dependence; (b) only used METH before they were incarcerated; (c) have been living a sober life for more than 1 month in the compulsory rehabilitation center; and (d) aged ≥18 years old. Exclusion criteria included the following: (a) history of mania, schizophrenia, or psychosis; (b) language difficulties; (c) vision or hearing impairments; (d) illicit drug use other than METH in the past; (e) any severe medical condition that may significantly affect brain and cardiovascular function; and 6) inability to tolerate the virtual reality helmet/environment. Current and lifetime diagnoses were determined by two experienced psychiatrists according to DSM‐V when the METH users were sent to the rehabilitation center. Besides, according to the Chinese law, the METH users need to be arrested by police at least twice before sending to the rehabilitation center. Those who were arrested the first time were sent to different institutions. The average length of METH use was 65.58 months (*SD* = 42.25). The average length since admitted to the rehabilitation center is 11.15 months (*SD* = 6.65).

The healthy control group included 332 male participants that matched the METH user group on age. All the healthy participants were recruited in Shandong and Beijing province through an online advertisement. They had no history of drug dependence or any mental problems. The exclusion criteria were the same as METH user group.

### 2.2 Virtual reality environment

**FIGURE 1:**
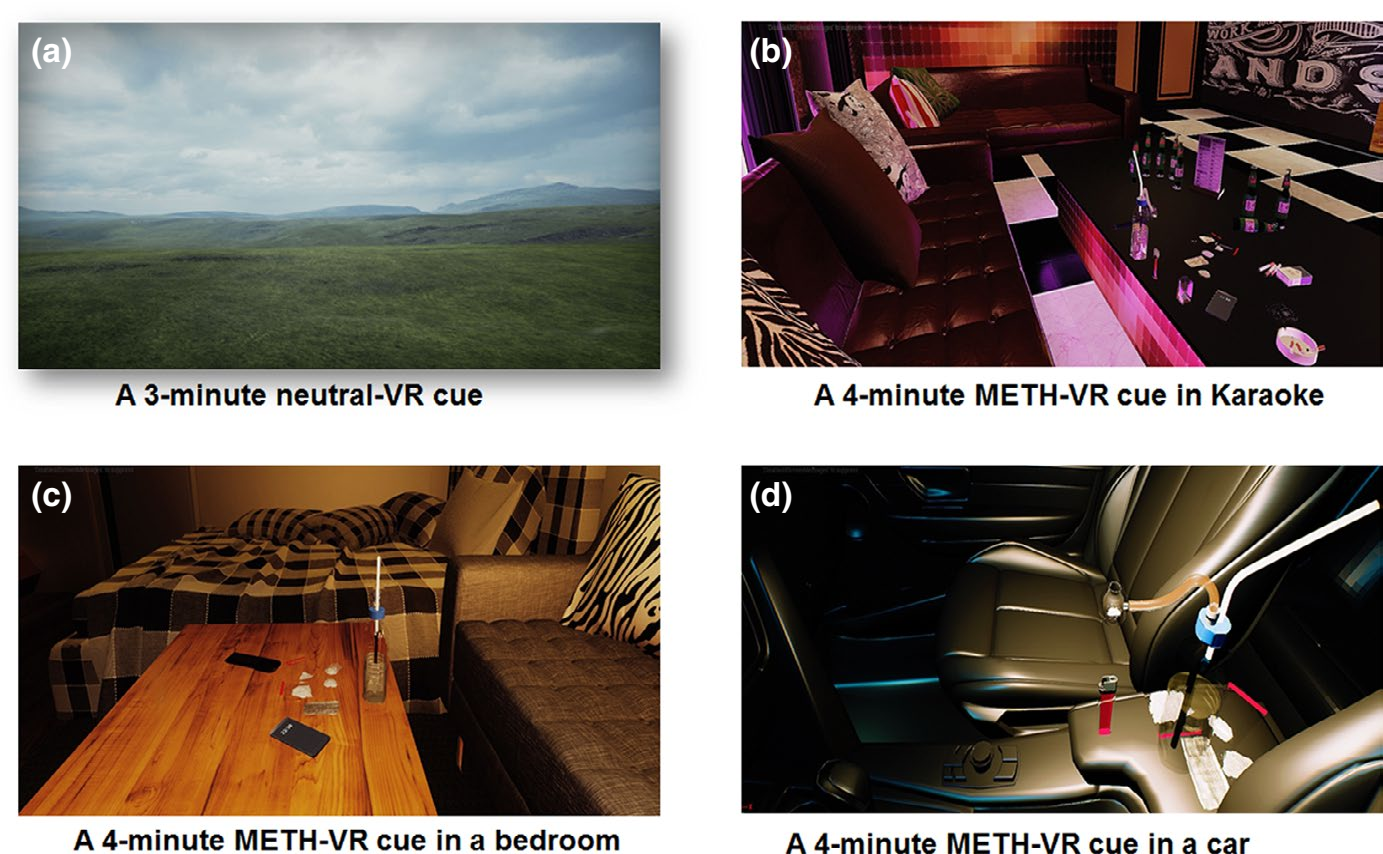
Screenshots of neutral‐VR cue and METH‐VR cue environment. (a) A 3‐min neutral scenario; (b) METH‐VR cue in karaoke; (c) METH‐VR cue in a bedroom; (d) METH‐VR cue in a car. METH, methamphetamine; VR, virtual reality

The virtual reality (VR) environment included two parts: neutral‐VR environment and METH‐VR environment. The neutral‐VR part was a 3‐min neutral grassland scenario, with clouds in the sky (Figure 1a). In this session, participants were required to be relaxed and look around in order to adapt to the VR environment. The METH‐VR environment included animate and auditory cues under three circumstances: in karaoke, in bedroom, and in a car. Each scenario lasted for 4 min (Figure 1b‐d), with avatars using METH and drug paraphernalia (e.g., glass pipe, medical tubing, and small plastic bags containing METH) in side (Culbertson et al., 2010). The participants were able to pick the drug paraphernalia in the VR environment by their hands and virtually use them. Auditory cues (e.g., snorting, smoking) appeared when they took the drugs in VR. The VR environment was presented by a VR helmet, with 2,560 × 1,440 pixels resolutions and 92 degrees field of view. The helmet was equipped with a custom‐built head tracker using a triaxial gyroscope, an accelerometer, and a compass sensor tracked at 1 MHz update rate. All participants were able to explore each VR scenario freely.

### 2.3 Data recording

EEG data were collected via a low‐cost, portable EEG headband (Adai‐jd‐001; Adai‐tech Co., Ltd.) at a sampling rate of 200 Hz with five electrodes located at Fpz, AF7, AF8, TP9, and TP10. Electrode Fpz was utilized as the reference electrode. EEG data on AF7, AF8, TP9, and TP10 were transmitted to a local server through Bluetooth. The raw data were filtered and processed by Brain Vision Analyzer software (Brain Products; GmbH) with a bandpass filter of 0.1 Hz–60 Hz. Using a fast Fourier transform, absolute power in 5 standard frequency bands of EEG were obtained: delta (1–4 Hz), theta (4–8 Hz), alpha (8–13 Hz), beta (13–30 Hz), and gamma (30–44 Hz).

Galvanic skin response (GSR) was recorded by a biofeedback unit (Grove‐GSR monitor; Mindfield Biosystem). Two sensors were affixed to the index and middle fingers of the participants’ nondominant hand. Data were transmitted to a local server by Bluetooth.

### 2.4 Feature selection

A classification analysis was conducted using the two input data modalities as input: EEG data (the absolute power values in 5 standard frequency bands) and GSR data. All the input data of the whole recording period were firstly mean‐centered and normalized prior to the data analysis. In order to explore the quick and convenient ways in classifying MEHT users from healthy controls, only the basic and common‐used features were selected in the present study. Mean and standard deviation (*SD*) of GSR and EEG powers in each VR scenario (one neutral‐VR cue and three METH‐VR cues) were calculated and used as the input features. The mean and *SD* of GSR reflect the changes in human sympathetic nervous system and are found useful in earlier studies (e.g., Healey & Picard, 2005; Kurniawan, Maslov, & Pechenizkiy, 2013). The mean and *SD* of absolute EEG power reflect the amount of EEG activity and its variation in one band independent of activity in other bands. Previous studies have proved that these two features were useful (e.g., Junghöfer, Elbert, Tucker, & Rockstroh, 2000; Oken & Chiappa, 1988). The total number of the features was 168 (4 scenarios × (2 variables [mean and *SD*] of GSR + 2 variables [mean and *SD*] × 4 EEG channels × EEG powers in 5 frequencies)). The data were labeled as drug abused patient or healthy subject. To evaluate and select the features, stepwise model selection and backward elimination method were used. AIC/BIC were used to choose model. A total of 56 features were selected eventually.

Three machine‐learning algorithms including random forest, logistic regression, and support vector machine (SVM) were used to build classification model. Random Forests is an ensemble model with a lot of decision trees. Each decision tree is trained with a dataset random sampled from the whole training set. The output of the method is the mode of the classification (Liaw & Wiener, 2002). Logistic regression aims at predicting a binary output value based on input variables. All input values are combined linearly. The coefficients of each input are optimized using gradient descent with cross‐entropy cost function (Fan, Chang, Hsieh, Wang, & Lin, 2008). SVM is also a common method to deal with linear and nonlinear classification issues in machine learning (Hearst, Dumais, Osman, Platt, & Scholkopf, 1998). Each input case was assigned to one category or the other in the SVM algorithm. The SVM training model is a representation of the input cases as points in space, mapped so that the two categories can be divided by a clear gap that is as wide as possible. New input cases are then predicted to belong to a category based on the side of the gap on which they fall.

To avoid overfitting, 80% of the total sample was included in the training process and the other 20% was included to test the accuracy, precision, sensitivity, and *f*1 score of each model. The *f*1 score is the harmonic average of the precision and sensitivity of a binary classification analysis. It ranges from 0 to 1, with higher scores indicating better performance of the machine‐learning model (Powers, 2011; Sakr et al., 2017). Furthermore, parameters for each algorithm were tested prior to finalize the machine‐learning models. The performance validation was then generated using 10‐fold cross‐validation and the accuracy, precision, sensitivity, and *f*1 score of the ten runs were averaged. Following previous studies (e.g., Ding et al., 2019; Friedrichs & Igel, 2005), the most commonly used tuning parameters were selected, including number of trees, minim samples at leaf, maximum depth of each tree, minimum samples at each node to split, and maximum features considered at each node for RF; regularization parameter C, tolerance to stop criteria, solver, and maximum iterations for LR; regularization parameter C, tolerance to stop criteria, and kernel for SVM. Feature importance analysis was done following information gain criterion.

### 2.5 Statistical analysis

Independent *t* test and chi‐square test were used to test whether there is a significant difference in age and educational background between METH user group and the healthy control group. A multivariate analysis of variance (MANOVA) was conducted to examine the differences in GSR and EEG power of 5 bands between METH user group and control group. A paired‐sample *t* test was conducted to test the differences in GSR and EEG power of 5 bands between neutral and METH‐VR scenarios.

All the machine‐learning analyses including classification and feature importance analysis were done by Jupyter Notebook (Project Jupyter). It supports scientific computing using Python. Python packages including Scikit‐learn (Pedregosa et al., 2011), NumPy, SciPy, and matplotlib were also utilized.

## 3 RESULTS

The demographic and clinical characteristics of the recruited group are displayed in Table 1. Independent *t* test showed no significant differences in age between the healthy control group (*m*ean age ± *SD*: 33.63 ± 7.92) and METH user group (*m*ean age 33.75 ± 6.49, *p* = .82). And chi‐square test showed that the two groups had no significant differences regarding educational background (χ2 = 0.15, *p* > .05).

**TABLE 1:** Demographic and clinical characteristics of the present sample

	METH (*n* = 333)	HC (*n* = 332)	*t*/χ^2^	*p*
Age (mean, *SD*)	33.75 (6.49)	33.63 (7.92)	0.22	.82
Gender	Male	Male	—	—
Education (*n*, %)	0.15	1.00		
Primary school	51 (15.3)	48 (14.5)		
Middle school	117 (35.1）	118 (35.5）		
High school	127 (38.1)	127 (38.3)		
College or above	38 (11.4)	39 (11.7)		

Abbreviations: administration length, the average length since being admitted to the isolated drug rehabilitation center (month); HC, healthy control participants; METH use length, the average length of METH use (month); METH, methamphetamine users.

### 3.1 Comparison of the METH user group and healthy comparison group

Table 2 shows mean EEG power in the 5 specific bands and mean GSR in the neutral and across the three METH‐VR scenarios. The MANOVA showed no interaction effects among the two subject groups and the four scenarios.

**TABLE 2:** Mean value and standard deviation of physiological data

	METH (*n* = 333)		HC (*n* = 332)		Interaction effects *F*(*p*)	Main effects *F* (*p*)	
	Neutral	METH‐VR	Neutral	METH‐VR	Group*Scenario	METH versus HC	Neutral versus MEHT‐VR
EEG							
delta	0.64 (0.22)	0.60 (0.22)	0.82 (0.26)	0.79 (0.25)	0.15 (*p* = .701）	105.32 (*p* < .001)	8.60 (*p* < .001)
theta	0.49 (0.20)	0.41 (0.18)	0.47 (0.23)	0.45 (0.22)	0.69 (*p* = .090）	0.77 (*p* = .087)	7.15 (*p* < .001)
alpha	0.48 (0.15)	0.46 (0.15)	0.51 (0.21)	0.51 (0.21)	1.00 (*p* = .318）	10.33 (*p* < .001)	4.50 (*p* < .001)
beta	0.59 (0.31)	0.57 (0.31)	0.50 (0.33)	0.50 (0.33)	0.32 (*p* = .569）	10.43 (*p* < .001)	0.32 (*p* = .569）
gamma	0.43 (0.35)	0.42 (0.35)	0.27 (0.39)	0.27 (0.39)	0.06 (*p* = .806）	30.13 (*p* < .001)	0.06 (*p* = .806)
GSR	2.43 (1.59)	2.46 (1.63)	5.01 (3.22)	5.10 (3.27)	0.05 (*p* = .831）	167.10 (*p* < .001)	4.47 (*p* < .001)

Note

Data format: mean (*SD*); EEG data were absolute power values.

Abbreviations: GSR, galvanic skin response; HC, healthy control group; METH, METH user group; METH‐VR, methamphetamine‐VR environment; neutral, neutral environment.

We took patient groups as the main effect, the GSR of METH user group was significantly lower than the healthy control subjects (*F* (323) = 167.10, *p* < .001). Since GSR is inversely correlated with skin conductance, the results revealed that METH user group showed greater stressful state. The EEG power in delta band (*F* (323) = 105.32, *p* < .001) and alpha band (*F* (323) = 10.33, *p* < .001) was significantly lower in METH group than the control group. The EEG power of beta band (*F* (323) = 10.43, *p* < .001) and gamma band ((*F* (323) = 30.13, *p* < .001) was significantly higher in METH group than the healthy group.

No significant differences were found in EEG power in theta band (*p* = .087).

While we test the differences between the VR scenarios (*Neutral* versus *METH‐VR*), the GSR of neutral‐VR was significantly higher than the METH‐VR (*t* (633) = 4.47, *p* < .001). The EEG power in delta (*t* (633) = −8.60, *p < .001*), theta (*t* (633) = −7.15, *p* < .001), alpha (*t* (633) = −4.50, *p < .001*) bands of neutral‐VR was significantly higher than the METH‐VR. No significant difference in beta and gamma bands was found between neutral‐VR and MRTH‐VR.

### 3.2 Results of classification

**FIGURE 2:**
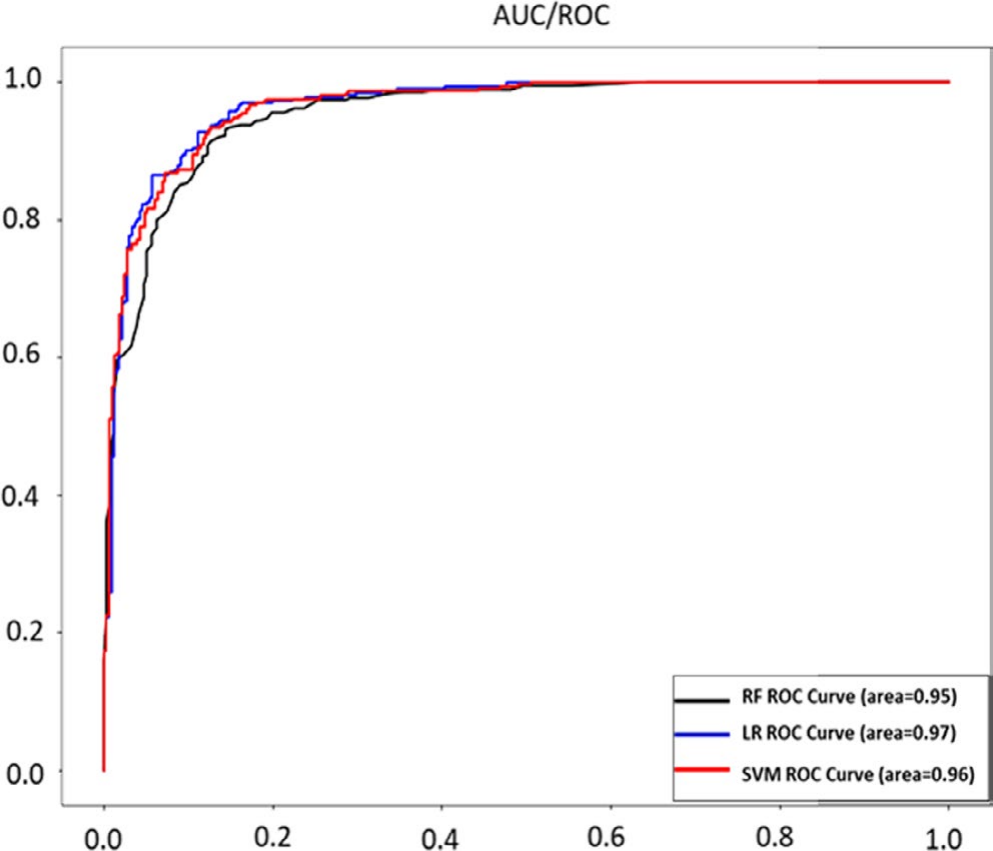
The area under the receiver operating characteristic curve (AUC/ROC) for the three classifiers. LR, logistic regression; RF, random forest; SVM, support vector machine

The indices of the two modalities (EEG and GSR) were combined and used as input to the classifiers. Table 3 shows the results of each classifier using testing dataset. Figure 2 showed the area under the receiver operating characteristic curve (AUC/ROC) for the three classifiers. The LR algorithm showed highest accuracy (90.68%) and F1 Score (90.80%). The parameters we used in each classifier are described here:

- For RF, number of trees = 100, minim samples at leaf = 1, maximum depth of each tree = 100, minimum samples at each node to split = 2, maximum features considered at each node = 65.
- For LR, regularization parameter C = 0.1, tolerance to stop criteria = 1e‐5, solver = 'newton‐cg', maximum iterations = 1,000.
- For SVM, regularization parameter C = 0.02, tolerance to stop criteria = 1e‐4, kernel = linear.

**TABLE 3:** The results of classifiers with EEG and GSR data as input

Classifier	Results (%)			
	Accuracy	Precision	Sensitivity	F1 score
RF	88.57 (86.00–91.14)	88.00 (83.06–92.94)	89.40 (87.53–91.27)	88.62 (86.04–91.20)
LR	**90.68 (88.72–91.61** **)**	**89.22 (86.22–92.22)**	**92.44 (91.17–93.71)**	**90.80 (88.72–88.72)**
SVM	90.38 (88.04–92.72)	88.27 (85.04–91.50)	93.01 (91.29–94.73)	90.56 (88.08–93.04)

Note

The range between the brackets is the confidence interval with 95%.

Abbreviations: LR, logistic regression; RF, random forest; SVM, support vector machine.

Bold numbers are indicates the classifier with the best performance.

Table 4 showed the top three variables and their importance index in each classifier based on the outcome of the feature selection process according to the information gain. The most important variables for LR classifier are as follows: mean of GSR in neutral scenario, *SD* of GSR in neutral scenario, and *SD* of GSR in METH‐VR (bedroom) scenario. It seems the mean GSR in neutral scenario is the most important variables in all the three classifiers. GSR gives more information in distinguishing methamphetamine‐dependent patients from healthy controls than EEG signals.

**TABLE 4:** The top significant three variables in each classifier

Classifier	Features	Importance
LR	1. Mean of GSR in neutral scenario	0.6564
	2. *SD* of GSR in neutral scenario	0.5762
	3. *SD* of GSR in METH‐VR (bedroom) scenario	0.4873
RF	1. Mean of GSR in neutral scenario	0.0998
	2. *SD* of GSR in neutral scenario	0.0931
	3. *SD* of GSR in METH‐VR (bedroom) scenario	0.0901
SVM	1. Mean of GSR in neutral scenario	0.3659
	2. *SD* of GSR in neutral scenario	0.3547
	3. Mean of alpha band in TP10 in METH‐VR (bedroom) scenario	0.2605

Abbreviations: GSR, galvanic skin response; LR, logistic regression; METH, methamphetamine; RF, random forest; *SD*, standard deviation; SVM, support vector machine.

## 4 DISCUSSION

Drug‐associated cues have been shown to elicit behavioral and physiological responses in patients with drug dependence (Ehrman, Robbins, Childress, & O’Brien, 1992; Franken et al., 2004). Reactivity to drug cues has been investigated as a possible indication of vulnerability to relapse (Drummond & Glautier, 1994). In this study, we proposed a method to distinguish patients with methamphetamine dependence from normal healthy subjects by comparing their reactions to drug‐associated cues. Culbertson et al demonstrated the usefulness of VR cues for eliciting subjective craving in METH abusers and showed its advantages versus. video cues (Culbertson et al., 2010). Therefore, we employed METH‐VR cues in this study.

The skin gives away lots of information on how we feel when we are exposed to emotionally loaded images, videos, events, or other stimulus. The galvanic skin response reflects the activity of sweat glands and the autonomous nervous system (ANS) as a whole. It generally reflects the skin's ability to transmit sweat enhanced electrical current. Fatseas et al. found a significant decrease of the galvanic skin response after drug‐related stimuli in drug relapsers (Fatseas et al., 2011). Our data also showed decreased galvanic skin response in METH‐abused group. Skin response decreases when skin conductance increases in more stressful and excited state. In a more relaxed state, skin response increases. The result of this study supports the excited state induced by METH (Ohme, Reykowska, Wiener, & Choromanska, 2009).

Prolonged drug use can have profound effects upon normal brain activity which can be recorded and measured through the use of quantitative EEG (qEEG) techniques. For example, previous studies have found that a majority of the conventional EEGs of the METH users were abnormal and METH users showed increased EEG power in the delta bands (Newton et al., 2003), increased theta quantitative EEG power on tasks that were more difficult (Newton et al., 2004), decreased cortical complexity of METH users (Yun et al., 2012), and higher clustering coefficient at the gamma band (Ahmadlou, Ahmadi, Rezazade, & Azad‐Marzabadi, 2013). In our study, the patients with methamphetamine dependence showed higher EEG power in gamma band and smaller EEG power in lower frequency bands, including delta (*p* < .001) and alpha (*p* < .001) frequency bands. These findings are very interesting. Gamma oscillations (>30 Hz) in the brain are involved in attention, perception, and memory. They are altered in various pathological states, as well as by neuropharmaceuticals. The changes of EEG power in different bands might suggest that methamphetamine abuse is associated with brain function deficits.

Machine‐learning methods have been widely tried in medical fields to predict different outcomes. This study is designed to take advantage of the unique database of patients with methamphetamine dependence, collected in a drug rehabilitation center to investigate the relative performance of various machine‐learning classification method for distinguishing normal subjects and patients with methamphetamine dependence by using EEG and GSR. To our knowledge, this is the first study using machine‐learning method in patients with methamphetamine dependence. We evaluated three machine‐learning methods, that is, SVM, RF, and LR, and there was not huge difference between their accuracy. In previous studies, Vomlet used five machine‐learning techniques (i.e., LR, Decision Tree, Naïve Bayes classifier, Artificial Neural Network, and Bayesian Network classifier) to predict mortality in patients with ST elevation myocardial infarction and he found the LR achieved the highest area under curve (Vomlel, Kruzık, Tuma, Precek, & Hutyra, 2012). In the present study, we achieved the highest *f*1 score by LR with a combination of EEG and GSR data (accuracy: 90.68%, precision: 89.22%, sensitivity: 92.44%, *f*1 score 90.08%). Previous studies that used machine‐learning models to classify patients (e.g., major depression patients) from healthy controls with EEG and GSR data achieved an average *f*1 score around 75%‐85% (e.g., Ding et al., 2019). The high *f*1 score in the present study indicates that the model would be useful clinically and has good potential in predicting METH users.

One of the common problems in machine learning is overfitting, which occurs when the model fits the peculiarities of the training dataset too much and does not find a general predictive rule (Dietterich, 1995). In order to avoid this problem, the present study used 80% of the total sample to train the models and used the other 20% of the total sample to test the accuracy, precision, sensitivity, and *f*1 score of each model. Therefore, the 20% of the sample that were used to test the performance of the models is independent from the training dataset. The high *f*1 score indicated a good generalization performance of the learned models. However, in order to further test the generalizability of the learned models, more studies with different samples are needed.

The present study is limited in several perspectives. First, only male drug users were included in this study; therefore, the machine‐learning model should be treated with caution when applies to female. Since male and female drug users were accommodated separately in China, multicenter studies should be considered in the future. The second limitation refers to the fact the present study did not include poly drug use. It is possible that poly drug users may have different cognitive pattern compared to those who only used METH. Moreover, future studies should consider comparing other drug‐dependent patients (e.g., cocaine) with methamphetamine‐dependent patients and further examine if the EEG pattern is methamphetamine specific. Third, the present study mainly focused on exploring whether machine learning approach can be used to distinguish METH users and healthy controls with EEG and GSR data. In order to use the outcomes as predictors to detect craving, further studies are needed with the measurement of METH craving and other covariates such as treatment compliance. Furthermore, there is the possibility that the METH group may come to look more like the healthy control group after a year of sobriety and treatment. Further studies with different sample (e.g., METH users who have not received any treatment) are needed in order to provide better understanding regarding this issue.

## 5 CONCLUSION

The study shows the potential of machine‐learning methods for distinguishing methamphetamine‐dependent patients from healthy subjects by using EEG and GSR data. The linear regression algorithm shows the best performance comparing with SVM and Forest Random.

## CONFLICT OF INTEREST

Dai Li and Yuanhui Li are working in Adai‐tech Company, which provides us with the equipment for data collection.

## AUTHOR CONTRIBUTIONS

The concept and study design were formed by XF.D., D.L., and XY.L.. Data acquisition and analysis were conducted by YH.L., D.L., and XF.D. Data explanation was conducted by XY.L., C.L.L, and XF.D. Drafting of the manuscript and figures was contributed by XY.L., XF.D., D.L., YH.L, and CL.L.

### Peer Review

The peer review history for this article is available at https://publons.com/publon/10.1002/brb3.1814.

## Data Availability

The data are available on reasonable request to corresponding author, Dr. Xiuyun Liu (liuxiuyun1@gmail.com).
